# Do aluminum (Al)-hyperaccumulator and phosphorus (P)-solubilising species assist neighbouring plants sensitive to Al toxicity and P deficiency?

**DOI:** 10.3389/fpls.2024.1371123

**Published:** 2024-04-24

**Authors:** M. Delgado, P. J. Barra, G. Berrios, M. L. Mora, P. Durán, A. Valentine, M. Reyes-Díaz

**Affiliations:** ^1^ Center of Plant, Soil Interaction and Natural Resources Biotechnology, Scientific and Technological Bioresource Nucleus (BIOREN), Universidad de La Frontera, Temuco, Chile; ^2^ Biocontrol Research Laboratory, Universidad de La Frontera, Temuco, Chile; ^3^ Departamento de Ciencias Químicas y Recursos Naturales, Facultad de Ingeniería y Ciencias, Universidad de La Frontera, Temuco, Chile; ^4^ Facultad de Ciencias Agropecuarias y Medioambiente, Departamento de Producción Agropecuaria, Universidad de La Frontera, Temuco, Chile; ^5^ Department of Horticultural Sciences, Faculty of AgriSciences, University of Stellenbosch, Stellenbosch, South Africa

**Keywords:** cluster roots, facilitation, nutrients, Proteaceae, *Gevuina avellana*, highbush blueberry

## Abstract

We aimed to evaluate the facilitation effects of an aluminum (Al) hyperaccumulator species bearing cluster roots, *Gevuina avellana*, on the seedling growth and performance of an Al-intolerant and phosphorus (P)-deficient-sensitive plant, *Vaccinium corymbosum*. For this, seedlings of *G. avellana* and *V. corymbosum* were grown alone or together as follows: i) two *G. avellana* seedlings, ii) one *G. avellana* + one *V. corymbosum* and iii) two *V. corymbosum*, in soil supplemented with Al (as Al_2_(SO_4_)_3_) and in the control (without Al supplementation). We determined relative growth rate (RGR), photosynthetic rate, chlorophyll concentration, lipid peroxidation and Al and nutrient concentration [Nitrogen (N), P, potassium (K), calcium (Ca), magnesium (Mg), sodium (Na), manganese (Mn), iron (Fe), copper (Cu), zinc (Zn), and sulfur (S)] in leaves and roots of both species. The results showed that, in general, *G. avellana* did not assist *V. corymbosum* to enhance its RGR nor reduce its Al uptake. However, *G. avellana* assisted *V. corymbosum* in enhanced N acquisition and, consequently, to increase its chlorophyll concentration and photosynthetic rate. Besides, *V. corymbosum* had lower lipid peroxidation in leaves when grown in the soil with high Al supplementation in association with *G. avellana.* Our results suggest a facilitating effect of *G. avellana* to *V. corymbosum* when grown in soils with high Al concentration, by enhancing chlorophyll concentrations and photosynthetic rate, and decreasing the oxidative damage to lipids.

## Introduction

Acidic soils (pH ≤ 5.5) are widely distributed, representing about 30% of the total world’s land area and, approximately 50% of the world’s potential arable lands. However, soil acidity is an important limitation for crop productivity. This is because, various restrictive factors for agricultural production usually coincide in these soils, such as toxic levels of aluminum (Al) and deficiencies in essential minerals for robust plant nutrition, such as phosphorus (P). Although Al is the third most abundant element in the earth’s crust, it lacks any discernible role in known biological processes (Tolrà et al., 2011). Under acidic conditions, the Al is released from soil particles into the soil solution as the trivalent ionic form (Al^3+^), which is particularly toxic for plants (Kochian et al., 2015). Aluminum rapidly hinders root growth, limiting both root expansion, and consequently constraining the absorption of water and essential nutrients vital for the plant’s fitness. Aluminum exists not only in the Al^3+^ ionic form but also is found as several insoluble forms including aluminosilicates, Al-humus complexes and other precipitated forms. Both the soluble and insoluble forms of Al can react and strongly retain minerals with negative charge, such as phosphate (H_2_PO_4_^-^, HPO_4_^2-^), decreasing its availability for plant nutrition (Kochian et al., 2004). Thus, both Al toxicity and P deficiency converge to exert a deep impact on plant growth and crop yields on acidic soils (Kochian et al., 2004; Chen and Liao, 2016).

In Southern Chile, soils from volcanic origin represent about 50-60% of arable land and support most of the agricultural production and forestry activities of the country (Borie and Rubio, 2003). The two main soil orders found in the southern-central Chile are Andisols and Ultisols, which inherently predisposed to natural acidity, harbor substantial amounts of active Al^3+^ and strong phosphate retention (Mora et al., 2006, 2017). Therefore, these soils are characterized by having high amounts of total P although with a very low P availability (Borie and Rubio, 2003). Consequently, traditional agricultural practices on acidic soils requires continuous application of agrochemicals, such as P fertilizers (a non-renewable resource) to increase P availability, and lime application (calcium carbonate) to increase soil pH and reduce Al phytotoxicity (Mora et al., 1999). However, these agricultural practices continued over time are unsustainable, since often they present negative environmental and socio-economic impact, and are not economically viable or physically manageable for many farmers worldwide (Samac and Tesfaye, 2003). Consequently, there is an increasing interest to develop environmentally friendly strategies to diminish our agrochemical dependency, and contribute to more sustainable agriculture.

Intercropping systems have proven to be an effective and sustainable alternative to counteract nutrient deficiency, along with increasing the use efficiency of fertilizers in the soil. This achievement can be attributed to the phenomenon of interspecific facilitation, encompassing both above- and belowground interactions (Michel et al., 2019; Schoebitz et al., 2020; Xu et al., 2020). Within belowground interactions, there are several studies evidencing that nutrient mobilization by the roots of some species plays an important role in the facilitation process, enhancing the nutrient uptake and growth of neighboring non-mobilizing species (Li et al., 2014; Lambers et al., 2018; Shen et al., 2023). In general, the nutrient mobilization is via root exudation of organic compounds, including phosphatases, organic acids, phenolic compounds, which increases the mineralization and solubilization of nutrients in the soil. The interspecific facilitation of nutrient acquisition by association of two or more plant species having different abilities to mobilize nutrient, have involved numerous species, including species bearing specialized root structures, such as cluster roots (Muler et al., 2013; Teste et al., 2014; Shen et al., 2023).

Cluster roots are described as ephemeral rootlets and root hairs around the central root forming structures type “brush” or “raceme” that actively exude organic compounds which play a pivotal role in efficiently mobilizing essential nutrients (Lambers et al., 2006; Lambers et al., 2015a). Many species have this type of root adaptation, including species that belong to Proteaceae family, which have been widely described as plants with remarkable ability to both acquire and utilize P efficiently (Lambers et al., 2015a). The nutrient-acquisition strategy of Proteaceae involves chemical modification of its rhizosphere through the exudates from their cluster roots, which release nutrients from the soil (Delgado et al., 2015; Lambers et al., 2015a). The primary exudates consist of organic acids which carry negative charges, enabling them to displace phosphate bound in the soil through interactions with cations such as Al^3+^, Fe^3+^ and Ca^2+^ (Ryan et al., 2001), making the previously bound phosphate readily available for plant uptake. Additionally, the organic acids chelate Al^3+^ to form stable and non-toxic complexes and, therefore, it is also involved in the detoxification of this metal (Kochian et al., 2015; Chen and Liao, 2016).

Certain cluster root-bearing species, such as several members of Proteaceae family have been classified as Al hyperaccumulator species. This means that they are able to accumulate high concentrations of Al within their leaves (≥ 1000 mg kg^−1^ dry weight) without manifesting any signs of stress or toxicity (Jansen et al., 2002). This intriguing trait suggests that the roots of these Al hyperaccumulator plants might absorb Al from their rhizosphere, potentially leading to a reduction of soil Al levels through continuous Al accumulation in leaves, bark and wood, as was early proposed by Webb (1954). Conversely, there are studies indicating that Al-hyperaccumulator species might acidify the rhizosphere, thereby increasing Al availability, as observed in *Camellia sinensis* L (Ruan et al., 2004; Chen et al., 2006). Despite these insights, our current understanding remains lacking in providing a comprehensive clarification regarding the influence of Al-hyperaccumulator species on soil Al availability, particularly within the context of species possessing cluster roots. Furthermore, the potential facilitative effect of an Al hyperaccumulator species, coupled with its ability to solubilize P, on a plant species sensitive to both Al toxicity and P deficiency, remains largely unexplored. In light of this, our study aimed to evaluate the effects of an Al hyperaccumulator species bearing cluster roots, *Gevuina avellana* Mol (Delgado et al., 2019), on the growth and performance of a neighboring species. For this investigation, we used *Vaccinium corymbosum* L., an economically important crop species, as a model species sensitive to both Al toxicity (Cárcamo-Fincheira et al., 2023) and P deficiency (Pinochet et al., 2014). Consequently, we posit that species with the dual ability to accumulate Al and solubilize P, may be suitable for the establishment and growth of neighboring plants in soils with high Al content and low P availability, such as volcanic soils in Southern Chile.

## Materials and methods

### Study species

*Gevuina avellana* is a native species from temperate rain forest of southern South America. This species belongs to the Proteaceae family, that typically produce cluster roots (**Supplementary Figure S1**), which exude large amounts of organic acids (Delgado et al., 2021). In their natural habitat, *G. avellana* grows in a wide range of soil conditions, whose P (Olsen) availability are in a range of 2.0 to 12.4 mg kg^-1^ (Delgado et al., 2018). In nursery conditions, this species can grow under nursery substrate having high P availability (22 mg kg^-1^) and it continues to forming cluster roots (Fajardo and Piper, 2019). Delgado et al. (2019) reported that *G. avellana* has the ability to accumulate large amounts of Al in its leaves (> 3,500 mg Al kg^-1^ dry weight) when growing under natural conditions. Thus, according to the criteria established by Jansen et al. (2002) *G. avellana* is considered an Al hyperaccumulating species (≥ 1,000 mg Al kg^-1^ in leaves).

*Vaccinium corymbosum* L. is an economically important crop cultivated in southern-central Chile for its world-famous and high-demand fruits (Montalba et al., 2019). Despite the fact that this species is adapted to acid soils, it is sensitive to Al^3+^ toxicity. The presence of Al^3+^ triggers a cascade of molecular, physiological and morphological changes in *V. corymbosum*, culminating in reduced productivity and yield (Reyes-Díaz et al., 2010, 2011; Inostroza-Blancheteau et al., 2011a, b; Inostroza-Blancheteau et al., 2013; Ulloa-Inostroza et al., 2016). Indeed, gypsum amendments are frequently used to ameliorate Al^3+^ toxicity in *V. corymbosum* (Alarcón-Poblete et al., 2019, 2020). According to the manual of fertilization for *V. corymbosum* cultivated in southern Chile (Pinochet et al., 2014), the maximum availability of exchangeable Al is 0.2 cmol^+^ kg^-1^, above which the *V. corymbosum* presents Al-toxicity. Likewise, according to Alarcón-Poblete et al. (2019), different cultivars of *V. corymbosum* presented leaf morphology alterations as well as photochemical and biochemical damages when grown in soil containing 22% of Al saturation. On the other hand, mineral fertilizers (including phosphate fertilizers) are required to ensure a better growth and yield of *V. corymbosum*. The optimal mineral content in soils for maximum performance of *V. corymbosum* depends on the mineralogical characteristics of the soil. Thus, in some regions of the world, the optimal available P content in the soil lies in a range between 30-60 mg kg^-1^ (Komosa et al., 2017; Ochmian et al., 2018). In contrast, the critical level for cultivating this species is 16 mg kg^-1^; below this value, *V. corymbosum* yield decline due to P deficiency (Pinochet et al., 2014). Therefore, we propose that the *V. corymbosum* is a good model species to study the facilitating effects of *G. avellana* under Al toxicity and P deficiency.

### Plant material

Plants of *G. avellana* and *V. corymbosum* were obtained from commercial nurseries. *Gevuina avellana* plants were cultivated from seeds, and were two years old when the experiment commenced, whereas *V. corymbosum* plants were produced *in vitro* and were one-year-old. At the beginning of the experiment, mean initial biomass (± s.e.) for *G. avellana* and *V. corymbosum* were 1.2 ± 0.8 and 2.6 ± 0.3 g, respectively. In this study, we used *V. corymbosum* cultivar Star because according to recent studies that evaluated the resistance to Al toxicity in different cultivars established in Chile, this cultivar emerged as the most Al-sensitive among the ones evaluated (Cárcamo et al., 2019; Cárcamo-Fincheira et al., 2023).

### Experimental design

In order to determine the facilitating effect of *G. avellana* on a sensitive species to Al toxicity and P deficiency, a greenhouse assay was carried out in the Universidad de La Frontera, Temuco, Chile. For this, soil from the series Freire, collected in Experimental Station Maquehue (38° 50′ 27″ S, 72° 41′ 39.03″ W) of the Universidad de La Frontera was used. To increase the Al in soil, we added Al sulfate (Al_2_(SO_4_)_3_), following the methodology reported by González-Villagra et al. (2021). We added 25 g of Al_2_(SO_4_)_3_ per 1.5 kg of soil, and after a month of soil incubation at room temperature (25° C approximately), we achieve values of exchangeable Al and percentage of Al saturation of 4.7 cmol+ kg^-1^ and 26.5%, respectively. Another part of the soil was not added with Al_2_(SO_4_)_3_ to be used as a control. The supplementation with Al_2_(SO_4_)_3_ to the soil shift the pH from ~5.7-6.2 down to 4.4-4.7, increasing significantly the availability of other elements such as Mn and S at the end of the experiment (**Table 1**).

**Table 1:** Soil chemical analysis of the experiment at the start and at the end of the experiment where *Gevuina avellana* and *Vaccinium corymbosum* were grown alone or in combination with or without aluminum sulfate supplementation in the following combinations: i) 2 seedlings of *G. avellana* (2Ga), 1 seedling of *G. avellana* + 1 seedling of *V. corymbosum* (1Ga + 1Vc), and iii) 2 seedlings of *V. corymbosum* (2Vc).

	Start	- (Al_2_(SO_4_)_3_)			Start	+ (Al_2_(SO_4_)_3_)		
		End of experiment				End of experiment		
		2Ga	1Ga+1Vc	2Vc		2Ga	1Ga+1Vc	2Vc
N (mg kg^-1^)	16.5 (1.4)	11.0 (0.4) a	12.5 (1.0) a	11.7 (1.8) a	14 (1.00)	11.3 (1.0) a	9.8 (1.2) a	8.8 (0.5) a
P-Olsen (mg kg^-1^)	18 (0.00)	16 (0.7) a	16 (0.0) a	11.7 (0.3) a	15 (0.00)	13.8 (0.3) a	13.3 (0.3) a	13.8 (0.3) a
K (mg kg^-1^)	174 (14.6)	102.6 (5.1) b	163.2 (4.3) a	180.8 (19.5) a	195.5 (2.3)	110.5 (8.0) b	114.4 (6.3) b	145.6 (10.0) a
pH (H_2_O)	5.7 (0.12)	6.2 (0.0) a	6.1 (0.0) a	6.0 (0.1) a	4.4 (0.02)	4.7 (0.0) b	4.7 (0.0) b	4.7 (0.0) b
Organic Matter (%)	15.5 (0.3)	14.3 (0.3) a	14.8 (0.3) a	14.5 (0.3) a	14.7 (0.33)	14.3 (0.3) a	14.3 (0.3) a	14.5 (0.3) a
K (cmol_+_ kg^-1^)	0.4 (0.04)	0.3 (0.0) b	0.4 (0.0) a	0.5 (0.0) a	0.5 (0.01)	0.3 (0.0) b	0.3 (0.0) b	0.4 (0.0) a
Na (cmol_+_ kg^-1^)	0.1 (0.03)	0.7 (0.0) ab	0.7 (0.0) b	0.7 (0.1) ab	0.2 (0.00)	0.7 (0.0) ab	0.8 (0.0) ab	0.9 (0.0) a
Ca (cmol_+_ kg^-1^)	9.3 (0.31)	9.2 (0.3) b	8.3 (0.2) b	8.4 (0.5) b	11 (0.07)	11.8 (0.3) a	11.1 (0.2) a	11.5 (0.4) a
Mg (cmol_+_ kg^-1^)	1.4 (0.02)	1.5 (0.0) a	1.4 (0.0) ab	1.4 (0.1) ab	1.5 (0.01)	1.4 (0.0) ab	1.3 (0.0) b	1.5 (0.0) a
Al (cmol_+_ kg^-1^)	0.1 (0.01)	0.1 (0.0) b	0.1 (0.0) b	0.1 (0.0) b	4.7 (0.07)*	1.6 (0.0) a	1.8 (0.0) a	1.9 (0.0) a
Al saturation (%)	0.5 (0.4)	0.5 (0.0) b	0.6 (0.0) b	0.7 (0.1) b	26.5 (0.39)*	10.0 (0.3) a	11.7 (0.3) a	11.7 (0.4) a
Mn (mg kg^-1^)	n.d.	7.2 (0.3) b	7.6 (0.5) b	6.1 (0.4) b	8.7 (0.3)	18.9 (0.6) a	18.0 (1.0) a	14.9 (1.7) a
S (mg kg^-1^)	n.d.	28 (1.3) b	27 (0.3) b	25 (2.6) b	n.d.	625 (10) a	600 (0.0) a	619 (6.3) a
*ECEC (cmol^+^ kg^-1^)	11.4 (0.3)	11.7 (0.0) b	10.9 (0.3) b	11.1 (0.6) b	17.8 (0.01)	15.7 (0.3) a	15.2 (0.2) a	16.0 (0.4) a
Sum of cations (cmol^+^ kg^-1^)	11.3 (0.3)	11.7 (0.3) b	10.9 (0.3) b	11.0 (0.6) b	13.1 (0.08)	14.2 (0.3) a	13.5 (0.2) a	14.3 (0.4) a

^1^ECEC, effective cation-exchange capacity. Each value corresponds to a mean of 3 samples ± standard error in brackets. Different letters indicate significant differences among treatments at the end of the experiment (*P* ≤ 0.05). * Indicate significant differences between treatments at the start the experiment (*P*< 0.01).

In this study, we conducted a greenhouse experiment in which both *G. avellana* (n = 60) and *V. corymbosum* (n = 60) individuals were subjected to two different conditions: i) the ‘Al supplemented soil’, where the soil was supplemented with Al_2_(SO_4_)_3_ and ii) the control, where no additional Al was added to the soil. The experiment utilized 4 L pots to accommodate the plants in the Al supplemented soil and the control, and they were allowed to grow either alone or together in the following combinations: i) 2 seedlings of *G. avellana* (conspecific species, n=10 pots), ii) 1 seedling of *G. avellana* + 1 seedling of *V. corymbosum* (interspecific species, n=10 pots), and iii) 2 seedlings of *V. corymbosum* (conspecific species, n =10 pots). The experiment was carried out under greenhouse conditions for 16 months (see the plants at the end of the experiment in **Supplementary Figure S2**). During the experiment, plants were watered regularly with tap water according to their requirements. Temperatures inside the greenhouse fluctuated between -1.6 and 31°C for the autumn-winter season (mean temperature: 11°C), and between 6.3 – 36.6°C for the spring-summer season (mean temperature: 18°C). The maximum light intensity recorded at noon varied between 129 and 485 µmol m^–2^ s^–1^ for the autumn-winter season, and between 283 and 712 µmol m^–2^ s^–1^ spring-summer season. At the end of the experiment, the morphological, chemical and physiological responses of these species were analyzed.

### Morphological measurements

Relative growth rate in height (RGRh) and biomass (RGRb) were determined in all surviving plants (Plant survival was 80 to 100% per treatment). For RGRh, seedling height (H) was recorded for all seedlings at the beginning (H_Initial_) and at the end (H_Final_) of the experiment. The RGRh was calculated according to Barrow (1977), where RGR (cm cm^–1^ day^–1^)= (ln H_Final_ – ln H_Initial_)/(t), t being the time (days) between the initial and final height measurements. The same calculations were made for RGRb, where the initial biomass was the average biomass of 8 initial plants. For biomass determinations, the leaves, stem and roots (non-cluster roots and cluster roots) of the seedlings were separated and weighed fresh using an analytical balance (Radwag AS 220.R2 Plus, Poland). Subsequently, leaves samples were divided into two subsamples: one for lipid peroxidation analyses; and the other for biomass and chemical analyses. For biomass analyses, the fresh samples (subsamples in the case of leaves) were dried in an oven at 60°C for 48 h to obtain dry weight. The dry biomass of total leaves of each seedling were calculated as the total fresh weight corrected for the moisture content of the leaves biomass subsample. The biomass distribution was calculated according to the percentage of each organ with respect to the total dry biomass of the plant. The total biomass of each individual corresponds to the sum of the dry weights of the different organs of the plant.

### Photosynthetic performance

Two days before the plants were harvested, photosynthetic rate, transpiration rate and stomatal conductance were measured between 9 and 12 a.m. in mature leaves formed during the experiment of both species, *G. avellana* and *V. corymbosum.* Two photosynthetic measurements were made per plant and six biological replicates per treatment were determined. For this, we used a portable infra-red gas analyzer photosynthesis system (LI-6400, LI-COR Bioscience, Inc., Lincoln, Nebraska, US) using a broad-leaf cuvette (area 2.5 cm^2^) and controlled light source (500 µmol photons m^-2^ s^-1^), temperature (20°C) and external CO_2_ (360 ppm). We decided to use 500 µmol photons m^-2^ s^-1^ because, in the case of *V. corymbosum*, previous studies reported that this species reach their maximum photosynthetic response at this photosynthetic photon flux density (PPFD) (Reyes-Díaz et al., 2016; Petridis et al., 2018). In the case of *G. avellana*, prior to determining the photosynthesis rate, we performed photosynthesis curves in response to PPFD, and we found that at 500 μmol photons m^−2^ s^−1^ this species had already reached its maximum photosynthesis rate, not being photoinhibited at this light intensity (**Supplementary Figure S4**). Additionally, at the end of the experiment, photosynthetic pigments such as Chlorophyll a, Chlorophyll b and carotenoids were determined in mature leaves. For this, 0.1 g of fresh leaf sample was homogenized with 1 mL of ethanol 96%. Homogenized sample mixture was centrifuged for 13,000 rpm for 5 min at 4°C. The supernatant was separated and the same step is repeated with the precipitate and then the two supernatants are combined. The solution mixture was spectrophotometrically measured at 665 nm, 649 nm and 470 nm. The quantification of Chlorophyll a, Chlorophyll b, and carotenoids were determined according to Lichtenthaler and Wellburn (1983).

### Lipid peroxidation

At the end of the experiment, lipid peroxidation was evaluated by thiobarbituric acid reacting substance (TBARS) quantification in fresh leaves of *G. avellana* and *V. corymbosum* following the modified protocol by Du and Bramlage (1992) using a Multimodal Microplate Reader Synergy HTX (BIOTEK). For this, we used 8-10 biological replicates per treatment.

### Chemical measurements in leaves and roots

At the end of the experiment, leaves and roots of all surviving plants of *G. avellana* and *V. corymbosum* were washed with tap water and later dried at 60°C for 48 h (n = 8 -10 replicates per species and treatment). The dried samples were ground to a powder using a grinding machine made of stainless steel (Bioscientific instruments, MRC, UK), and ground samples were used to analyze macro- and micronutrients including P, nitrogen (N), sulfur (S), manganese (Mn), iron (Fe), copper (Cu), zinc (Zn), calcium (Ca), sodium (Na), potassium (K), magnesium (Mg) and Al. For concentrations of micronutrients (Na, Mn, Cu, Fe, and Zn) and some macronutrients (Ca, K and Mg) as well as Al, 0.5 g of sample were ashed at 500°C for 8 h. The resulting ash was digested using hydrochloric acid (2 M) as described in Sadzawka et al. (2004b) and the different elements were quantified using an atomic absorption spectrophotometer (GBC Scientific Equipment Pty Ltd., SavantAA, Sigma, Dandenong, Victoria, Australia). Phosphorus concentrations were determined spectrophotometrically using the vanado-phosphomolybdate method, while N concentrations were determined by Kjeldahl distillation after acidic digestion (Sadzawka et al., 2004b). For S concentration, leaves and roots were dried as mentioned above, treated with 95% magnesium nitrate (MgNO_3_ × 6H_2_O), and ashed at 500°C for 4 h. Ashed samples were digested in 10 mL of HCl (2 M) at 150°C for 60 min, filtered and graduated with deionized water to 50 mL. Subsequently, filtered samples were mixed with barium chloride (BaCl_2_) and Tween-80. The resulting solution was measured spectrophotometrically (Spectronic GenesysTM, NY) at 440 nm, as described by Sadzawka et al. (2004b).

### Chemical measurements in soil

Three soil samples per treatment were analyzed at the beginning and end of the experiment (Sadzawka et al., 2004a). Mineral N were determined using the Kjeldahl method according to Bremner (1960) using a Kjeldahl UDK129 distiller equipment (VELP Scientific, Italy). Soil P (Olsen) was determined colorimetrically by applying the phosphoantimonylmolybdenum blue complex method (Drummond and Maher, 1995). Organic matter was measured following the wet digestion method by Walkley and Black (1934). Exchangeable cations [potassium (K), sodium (Na), calcium (Ca), and magnesium (Mg)] and exchangeable Al were extracted according to Sadzawka et al. (2004a) and determined using an atomic absorption spectrophotometer (GBC Scientific Equipment Pty Ltd.). Percentage of Al saturation was calculated with respect to the total sum of the exchangeable cations (Ca^+^, K^+^, Na^+^, Mg^+^, Al^+^). Soil pH was determined in soil suspended in water (ratio 1:2.5; w/v H_2_O). Soil Mn concentration was determined using the diethylenetriamine pentaacetic acid (DTPA) method described by Lindsay and Norvell (1978) and measured by atomic absorption spectrophotometry (GBC Scientific Equipment Pty Ltd.).

Sulfur in soil was measured according the methodology by Sadzawka et al. (2004b). Briefly, sulfur was extracted using a Ca(H_2_PO_4_)_2_ 0.01 M solution, followed by a turbidimetric measurement of sulphate as BaSO_4_ and measured spectrophotometrically (Spectronic GenesysTM, NY) at 372 nm.

### Statistics

Statistical analyses were carried out separately for each species using a one-way ANOVA with Tukey’s posteriori test (P ≤ 0.05) for parameters related to growth (n = 8-10 replicates), physiological [photosynthetic rate, chlorophyll concentration, (n = 6 replicates)], biochemical [lipid peroxidation, (n = 8-10 replicates)] and chemical [nutrients and Al concentrations, (n = 8-10 replicates)] responses of *G. avellana* and *V. corymbosum* when grown alone or in combination with or without Al supplementation. All data passed the normality and equal variance tests. Statistical analyses were performed using the Sigma Plot v.12.

## Results

### Relative growth rate and biomass distribution

**Figure 1:**
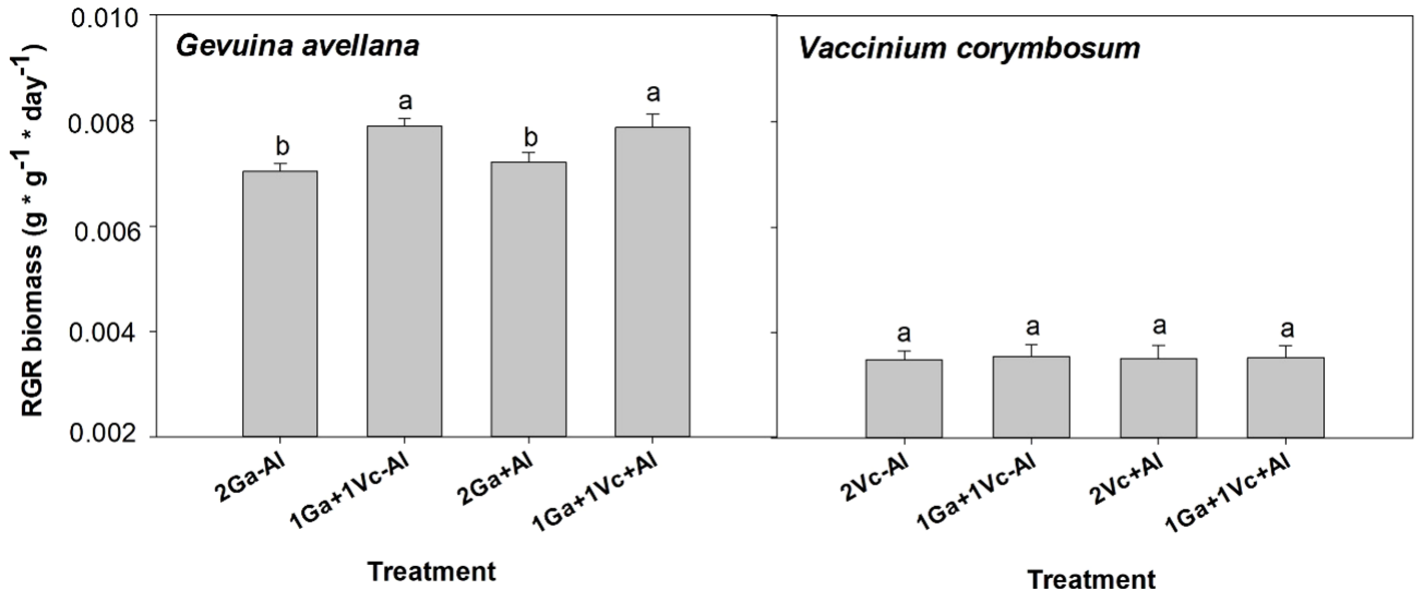
Relative growth rate (RGR) in biomass of *Gevuina avellana* and *Vaccinium corymbosum* growing alone or in combination with or without aluminum sulfate supplementation in the following combinations: i) 2 seedlings of *G. avellana* (2Ga), 1 seedling of *G. avellana* + 1 seedling of *V. corymbosum* (1Ga + 1Vc), and iii) 2 seedlings of *V. corymbosum* (2Vc). Each bar corresponds to mean of eight to ten samples ± standard error (SE). Different letters indicate significant differences among treatments (*P* ≤ 0.05).

The findings of this study revealed significant increases in the total biomass (**Supplementary Table S1**) and RGR in biomass for *G. avellana* when it grew together with *V. corymbosum* in both soil conditions, regardless of the presence or absence of Al (**Figure 1**). In contrast, similar RGR in biomass were recorded for *V. corymbosum* across all treatments, irrespective of soil condition and neighboring species (**Figure 1**). Upon analyzing the biomass allocation patterns, it was observed that *G. avellana* allocated approximately 48% of its biomass to leaves, 15% to the stem, 25% to non-cluster roots, and 12% to the formation of cluster roots. In contrast, *V. corymbosum* allocated its biomass differently, with approximately 16% directed to leaves, 23% to the stem, and the majority, 61%, to non-cluster roots (**Supplementary Figure S3**). These biomass allocations remained unaffected by the experimental treatments.

### Photosynthetic performance

**Figure 2:**
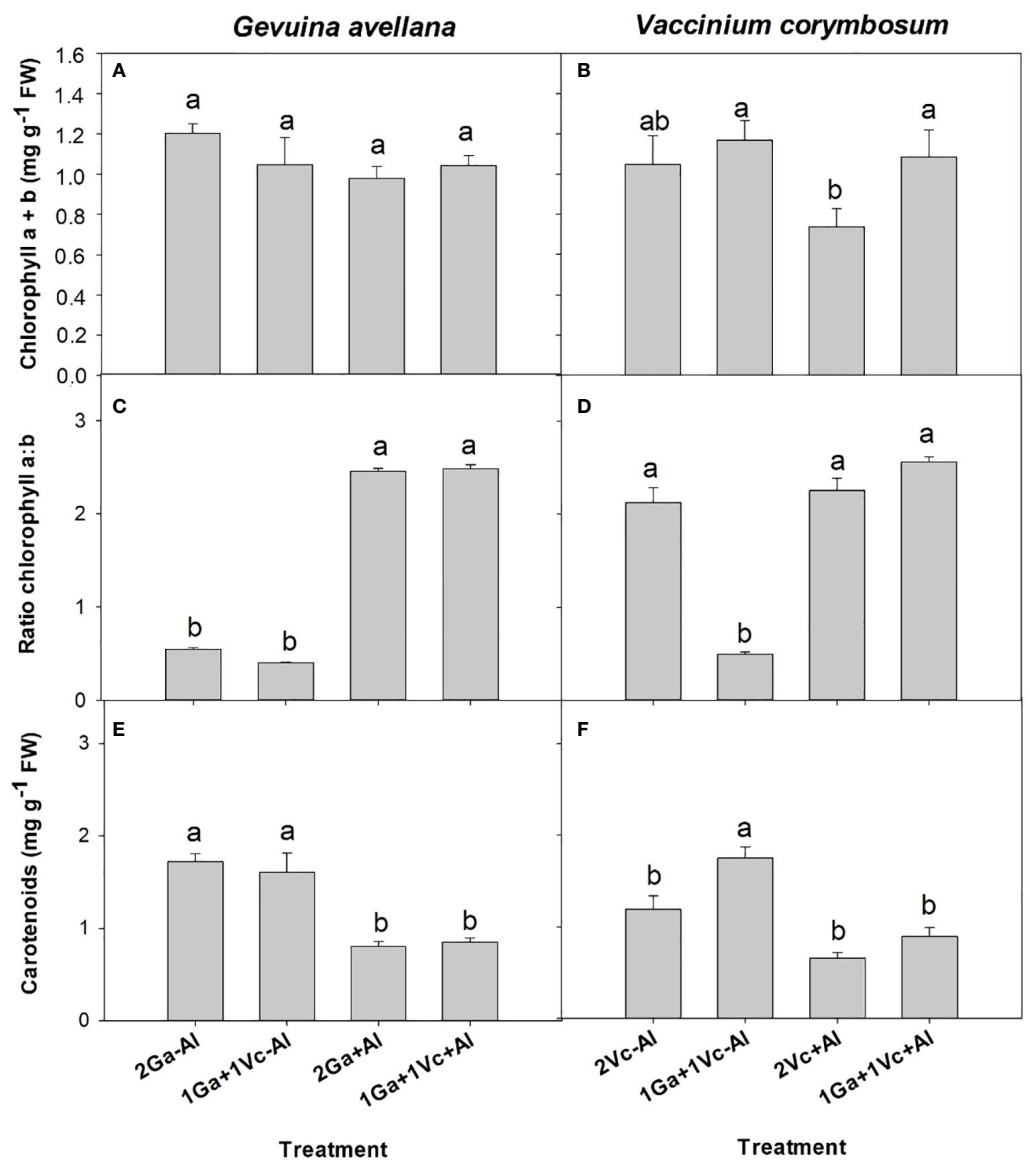
Chlorophyll a + b concentration, ratio chlorophyll a:b and total carotenoids of *Gevuina avellana* **(A, C, E)** and *Vaccinium corymbosum* **(B, D, F)** growing alone or in combination with or without aluminum sulfate supplementation in the following combinations: i) 2 seedlings of *G. avellana* (2Ga), 1 seedling of *G. avellana* + 1 seedling of *V. corymbosum* (1Ga + 1Vc), and iii) 2 seedlings of *V. corymbosum* (2Vc). Each bar corresponds to mean of six samples ± standard error (SE). Different letters indicate significant differences among treatments (*P* ≤ 0.05).

Although no significant differences were observed in the leaf chlorophyll (a + b) concentration of *G. avellana* plants (**Figure 2A**), this species showed a significantly higher photosynthetic rate when grown in Al-supplemented soil compared to control plants (**Table 2**). On the contrary, *V. corymbosum* presented a higher leaf chlorophyll concentration and photosynthetic rate in control soil conditions (**Table 2**, **Figure 2B**). Interestingly, *V. corymbosum* plants grown in Al-supplemented soil, significantly increased its photosynthesis rate and leaf chlorophyll concentration when grown together with *G. avellana* than when grown accompanied by a conspecific species (**Table 2**, **Figure 2B**). The ratio of chlorophyll a:b showed that *G. avellana* leaves presented significatively higher chlorophyll-a when grown in Al supplemented soil (**Figure 2C**). In leaves of *V. corymbosum* plants it was observed that the ratio of chlorophyll a:b was significantly lower in plants grown in the control soil but only when was accompanied by *G. avellana* (**Figure 2D**). The carotenoids concentration in *G. avellana* leaves decreased significantly when plants were grown in Al supplemented soil (**Figure 2E**), whereas in leaves of *V. corymbosum* the carotenoid concentration increased significantly in plants grown in the control soil but only when was accompanied by *G. avellana* (**Figure 2F**).

**Table 2:** Photosynthesis rate, transpiration rate and stomatal conductance of *Gevuina avellana* and *Vaccinium corymbosum* growing alone or in combination with or without aluminum sulfate supplementation in the following combinations: i) 2 seedlings of *G. avellana* (2Ga), 1 seedling of *G. avellana* + 1 seedling of *V. corymbosum* (1Ga + 1Vc), and iii) 2 seedlings of *V. corymbosum* (2Vc).

Treatment	Photosynthesis rate (µmol CO_2_ m^-2^ s^-1^)	Transpiration rate (mmol H_2_O m^-2^ s^-1^)	Stomatal conductance (mol H_2_O m^-2^ s^-1^)
*Gevuina avellana*			
2Ga-Al	4.4 (0.2) b	1.3 (0.03) n.s.	0.4 (0.01) b
1Ga+1Vc-Al	4.7 (0.1) b	1.4 (0.05) n.s.	0.4 (0.02) ab
2Ga+Al	5.3 (0.0) a	1.3 (0.05) n.s.	0.5 (0.01) a
1Ga+1Vc+Al	5.3 (0.1) a	1.5 (0.04) n.s.	0.5 (0.01) a
*Vaccinium corymbosum*			
2Vc-Al	5.4 (0.1) a	1.3 (0.03) b	0.4 (0.01) b
1Ga+1Vc-Al	5.4 (0.1) a	1.5 (0.05) a	0.5 (0.02) a
2Vc+Al	3.4 (0.2) c	1.1 (0.02) b	0.3 (0.02) c
1Ga+1Vc+Al	4.8 (0.1) b	1.5 (0.04) a	0.5 (0.02) a

Each value corresponds to mean of six samples ± standard error (SE). Different letters in each column indicate significant differences among treatments (*P* ≤ 0.05). n.s., There are no significant differences among treatments.

### Leaf N:P ratio

**Figure 3:**
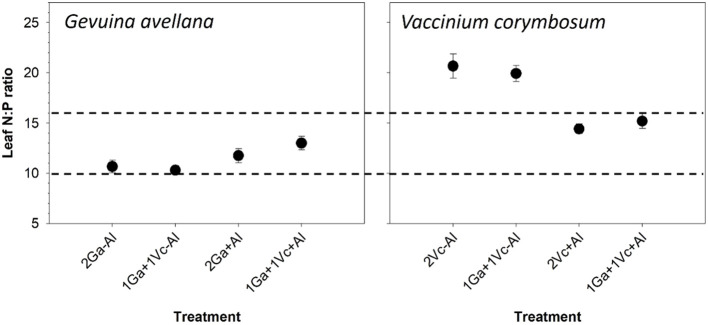
Leaf N:P ratio of *Gevuina avellana* and *Vaccinium corymbosum* growing alone or in combination with or without aluminum sulfate supplementation in the following combinations: i) 2 seedlings of *G. avellana* (2Ga), 1 seedling of *G. avellana* + 1 seedling of *V. corymbosum* (1Ga + 1Vc), and iii) 2 seedlings of *V. corymbosum* (2Vc). Each bar corresponds to mean of eight to ten samples ± standard error (SE). Different letters indicate significant differences among treatments (*P* ≤ 0.05).

The leaf N:P ratio served as a reliable indicator to assess whether N or P limitation influenced the plants, with values < 10 suggesting N limitation, values > 16 indicating P limitation, and values between 10 and 16 implying that plant growth was equally constrained by N and P (Koerselman and Meuleman, 1996). The results showed that *G. avellana* was co-limited by N and P in all treatments, whereas *V. corymbosum* was P limited in the control soil and co-limited by N and P when plants grown in Al supplemented soil (**Figure 3**).

### Lipid peroxidation

**Figure 4:**
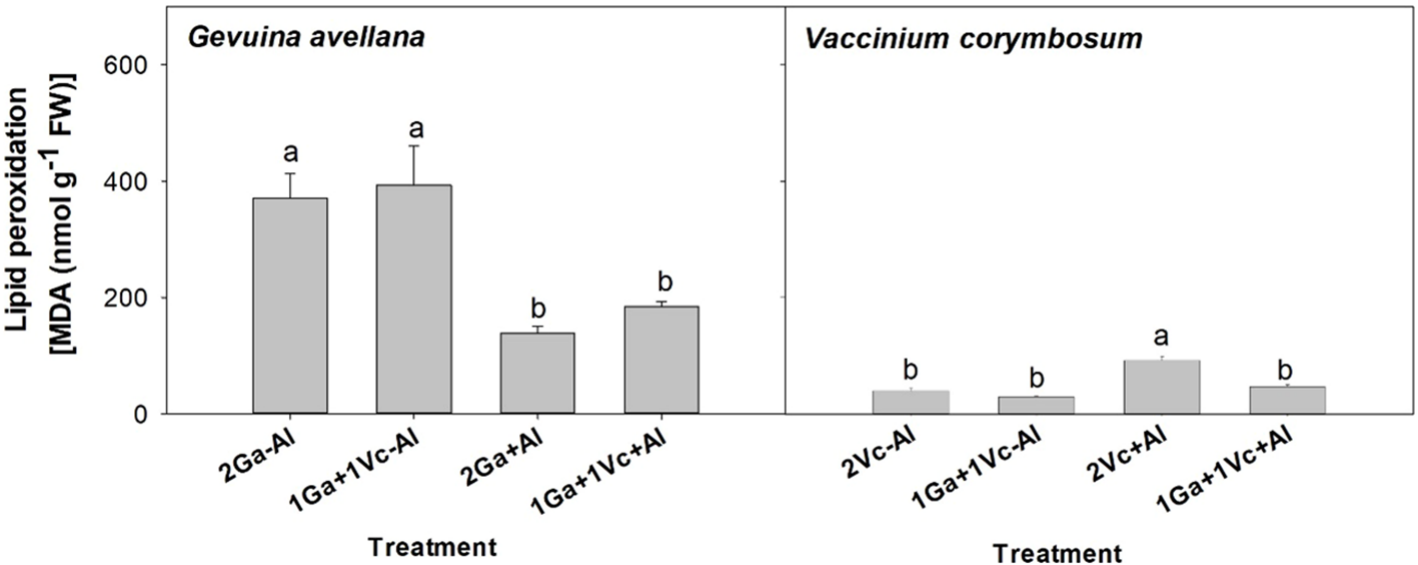
Lipid peroxidation of leaves of *Gevuina avellana* and *Vaccinium corymbosum* growing alone or in combination with or without aluminum sulfate supplementation in the following combinations: i) 2 seedlings of *G. avellana* (2Ga), 1 seedling of *G. avellana* + 1 seedling of *V. corymbosum* (1Ga + 1Vc), and iii) 2 seedlings of *V. corymbosum* (2Vc). Each bar corresponds to mean of eight to ten samples ± standard error (SE). Different letters indicate significant differences among treatments (*P* ≤ 0.05).

The lipid peroxidation of *G. avellana* leaves was higher in plants that grew in the control soil. On the contrary, higher lipid peroxidation was observed in the leaves of *V. corymbosum* plants that grew in the Al-supplemented soil and accompanied by another *V. corymbosum* plant (**Figure 4**).

### Leaves and roots nutrient concentration

The Al-supplemented soil significantly affected the nutrients uptake from the plants, depending on the species (**Tables 3**, **4**). For example, *G. avellana* maintains its levels of foliar and roots N concentration independent of the soil from which it grew and the neighboring species (conspecific or interspecific). In contrast, *V. corymbosum* presented lower N values in the Al supplemented soil, these being significantly lower when this species grows accompanied by a conspecific species than when it grows accompanied by *G. avellana.*

**Table 3:** Nitrogen (N), phosphorus (P), potassium (K), calcium (Ca), magnesium (Mg), sodium (Na), manganese (Mn), iron (Fe), copper (Cu), zinc (Zn), aluminum (Al) and sulfur (S) concentrations in leaves and roots of *Gevuina avellana* grown alone or in combination with or without aluminum sulfate supplementation in the following combinations: i) 2 seedlings of *G. avellana* (2Ga), 1 seedling of *G. avellana* + 1 seedling of *V. corymbosum* (1Ga + 1Vc), and iii) 2 seedlings of *V. corymbosum* (2Vc).

	*Gevuina avellana* (Leaves)			
	- (Al_2_ (SO_4_)_3_)		+ (Al_2_ (SO_4_)_3_)	
	2Ga	1Ga+1Vc	2Ga	1Ga+1Vc
N (mg g^-1^)	10.1 (0.21) a	10.6 (0.45) a	9.6 (0.37) a	10.3 (0.64) a
P (mg g^-1^)	0.99 (0.06) a	1.06 (0.07) a	0.75 (0.06) b	0.89 (0.06) ab
K (mg g^-1^)	2.9 (0.1) c	3.7 (0.2) b	7.1 (0.6) a	8.54 (0.9) a
Ca (mg g^-1^)	7.03 (0.21) b	6.4 (0.3) b	7.5 (0.4) ab	9.3 (1.0) a
Mg (mg g^-1^)	0.96 (0.03) b	1.0 (0.1) b	3.2 (0.3) a	2.8 (0.2) a
Na (mg g^-1^)	1.13 (0.6) b	1.0 (0.1) b	2.5 (0.2) a	2.7 (0.4) a
Mn (mg kg^-1^)	552 (31.3) b	532 (38.6) b	1161 (95) a	1286 (170) a
Fe (mg kg^-1^)	95.0 (6.8) a	74.8 (4.1) ab	65.9 (2.2) b	88.4 (14.09) ab
Cu (mg kg^-1^)	2.74 (0.14) b	2.6 (0.2) b	3.2 (0.18) ab	3.9 (0.2) a
Zn (mg kg^-1^)	12.3 (0.8) a	12.2 (1.0) a	15.2 (1.1) a	13.0 (0.8) a
Al (mg kg^-1^)	1501 (80.7) a	1599 (142) a	1325 (82) a	1594 (149) a
S (mg g^-1^)	0.9 (0.0) b	1.00 (0.1) b	11.9 (1.0) a	10.5 (0.7) a
	*Gevuina avellana* (Roots)			
	- (Al_2_ (SO_4_)_3_)		+ (Al_2_ (SO_4_)_3_)	
	2Ga	1Ga+1Vc	2Ga	1Ga+1Vc
N (mg g^-1^)	9.8 (0.2) a	10.6 (0.3) a	10.7 (0.6) a	9.2 (0.4) a
P (mg g^-1^)	0.20 (0.00) ab	0.23 (0.01) a	0.19 (0.01) b	0.22 (0.01) a
K (mg g^-1^)	2.48 (0.12) b	2.81 (0.33) b	4.10 (0.36) ab	5.94 (0.39) a
Ca (mg g^-1^)	4.19 (0.10) a	4.22 (0.10) a	3.72 (0.14) a	3.63 (0.28) a
Mg (mg g^-1^)	1.25 (0.04) b	1.32 (0.06) b	1.37 (0.06) ab	1.62 (0.13) a
Na (mg g^-1^)	1.80 (0.10) b	1.89 (0.21) b	2.11 (0.10) b	2.79 (0.19) a
Mn (mg kg^-1^)	92.9 (6.0) b	152.3 (14.6) b	334.8 (20.7) a	364.7 (39.34) a
Fe (mg kg^-1^)	1768 (130) b	3859 (444) a	3334 (404) a	2738 (347) ab
Cu (mg kg^-1^)	17.55 (1.6) b	22.43 (2.3) ab	29.5 (2.2) a	23.1 (1.3) ab
Zn (mg kg^-1^)	12.7 (0.9) b	16.8 (1.2) ab	31.2 (6.8) a	16.8 (0.7) ab
Al (mg k^1^)	3775 (205) b	7026 (692) a	8277 (639) a	7882 (541) a
S (mg g^-1^)	0.99 (0.06) b	1.17 (0.04) b	5.7 (0.4) a	5.0 (0.2) a

Each value corresponds to a mean of eight to ten samples ± standard error in brackets. Different letters indicate significant differences among treatments (*P* ≤ 0.05).

**Table 4:** Nitrogen (N), phosphorus (P), potassium (K), calcium (Ca), magnesium (Mg), sodium (Na), manganese (Mn), iron (Fe), copper (Cu), zinc (Zn), aluminum (Al) and sulfur (S) concentrations in leaves and roots of *Vaccinium corymbosum* grown alone or in combination with or without aluminum sulfate supplementation in the following combinations: i) 2 seedlings of *G. avellana* (2Ga), 1 seedling of *G. avellana* + 1 seedling of *V. corymbosum* (1Ga + 1Vc), and iii) 2 seedlings of *V. corymbosum* (2Vc).

	*Vaccinium corymbosum* (Leaves)			
	- (Al_2_ (SO_4_)_3_)		+ (Al_2_ (SO_4_)_3_)	
	2Vc	1Ga+1Vc	2Vc	1Ga+1Vc
N (mg g^-1^)	11.1 (0.4) a	10.7 (0.2) a	8.5 (0.2) b	10.5 (0.4) a
P (mg g^-1^)	0.56 (0.02) a	0.54 (0.02) a	0.63 (0.02) a	0.66 (0.03) a
K (mg g^-1^)	7.0 (0.2) b	7.7 (0.3) b	13.2 (0.4) a	13.7 (0.8) a
Ca (mg g^-1^)	6.6 (0.3) b	7.6 (0.5) ab	7.9 (0.3) a	8.7 (0.6) a
Mg (mg g^-1^)	1.8 (0.1) b	2.1 (0.1) b	3.2 (0.1) a	3.4 (0.4) a
Na (mg g^-1^)	1.0 (0.1) a	1.2 (0.1) a	0.9 (0.1) ab	0.7 (0.1) b
Mn (mg kg^-1^)	78 (5.9) c	123 (14.8) b	717 (37) a	718 (86) a
Fe (mg kg^-1^)	114 (9.6) a	139 (12.8) a	107 (13.3) a	81 (5.3) a
Cu (mg kg^-1^)	3.2 (0.2) b	4.0 (0.3) ab	3.3 (0.12) ab	4.3 (0.3) a
Zn (mg kg^-1^)	23.8 (1.1) a	21.8 (1.3) ab	16.3 (0.7) c	18.0 (1.2) bc
Al (mg kg^-1^)	157 (18.2) a	196 (26.6) a	142 (21.1) a	121 (9.3) a
S (mg g^-1^)	0.48 (0.03) c	0.62 (0.04) b	8.2 (0.4) a	8.5 (0.9) a
	*Vaccinium corymbosum* (Roots)			
	- (Al_2_ (SO_4_)_3_)		+ (Al_2_ (SO_4_)_3_)	
	2Vc-Al	1Ga+1Vc-Al	2Vc+Al	1Ga+1Vc+Al
N (mg g^-1^)	9.9 (0.3) a	9.6 (0.21) a	8.67 (0.26) b	8.7 (0.2) ab
P (mg g^-1^)	0.87 (0.04) a	0.25 (0.02) b	0.26 (0.01) b	0.28 (0.03) b
K (mg g^-1^)	2.78 (0.11) b	2.60 (0.22) b	2.59 (0.11) b	3.16 (0.14) a
Ca (mg g^-1^)	3.51 (0.15) a	3.21 (0.42) a	3.69 (0.27) a	2.93 (0.27) a
Mg (mg g^-1^)	1.24 (0.03) b	1.23 (0.17) b	1.63 (0.04) a	1.84 (0.07) a
Na (mg g^-1^)	0.50 (0.02) a	0.56 (0.08) a	0.43 (0.02) b	0.51 (0.05) a
Mn (mg kg^-1^)	172 (14.6) c	225 (26.4) c	580 (30.2) b	743 (45.3) a
Fe (mg kg^-1^)	3543 (425) a	4008 (862) a	3703 (399) a	4723 (731.8) a
Cu (mg kg^-1^)	25.8 (2.2) b	28.7 (5.6) ab	28.8 (1.9) ab	37.5 (3.4) a
Zn (mg kg^-1^)	17.3 (0.9) a	20.2 (4.3) a	20.0 (0.8) a	23.4 (2.3) a
Al (mg kg^-1^)	5723 (556) a	6411 (820) a	8022 (605) a	8395 (958) a
S (mg g^-1^)	1.13 (0.05) b	1.12 (0.04) b	3.93 (0.26) a	3.78 (0.3) a

Each value corresponds to a mean of eight to ten samples ± standard error in brackets. Different letters indicate significant differences among treatments (*P* ≤ 0.05).

The leaf and roots P concentration was significantly lower in *G. avellana* plants that grew in the Al supplemented soil and when grown with a conspecific species (**Table 3**). In contrast, *V. corymbosum* did not show significant differences in foliar P concentration in the different treatments evaluated, although it did show a significantly higher P concentration in roots when it grew in control soil and accompanied by a conspecific species (**Table 4**).

In general, mineral nutrients such as K, Mg and Mn significantly increased their concentration in the leaves and roots of both species when they were grown in the Al supplemented, regardless of whether the neighboring species was interspecific or conspecific. Similar trend was found in Na concentration in leaves and roots of *G. avellana* (**Table 3**). However, Na concentration in leaves and roots of *V. corymbosum* tends to decrease with Al supplementation, being significantly lower in the leaves of *V. corymbosum* plants that grew together with an interspecific species and in the roots of plants that grew together with a conspecific species (**Table 4**).

The concentration of Ca and Cu in the leaves of *G. avellana* and *V. corymbosum* also increased with Al supplementation, however, this was only significantly higher when both species were accompanied by an interspecific species. No significant differences were found in roots Ca concentration of both species in the different treatments evaluated (**Tables 3**, **4**).

The Zn concentration in leaves and roots varied depending on the species and the treatment. First, *G. avellana* did not present significant differences in leaf Zn concentration, but root Zn concentration increased when the plants grew with Al supplementation and when they were accompanied by a conspecific species (**Table 3**). On the contrary, in *V. corymbosum* plants a decrease in the leaf Zn concentration was observed in the soil supplemented with Al, being significantly lower when the plants grew accompanied by a conspecific species (**Table 4**).

No significant differences were observed in Fe concentration in leaves and roots of *V. corymbosum* plants among the evaluated treatments (**Table 4**). On the contrary, *G. avellana* presented a decrease in leaf Fe concentration when the plants grew in the Al supplemented soil and accompanied by a conspecific species compared to the control plants. On the other hand, an increase in roots Fe concentration was observed in *G. avellana* plants in all the treatments compared to the plants that grew in the control soil and accompanied by a conspecific species (**Table 3**).

In both species, leaf Al concentration was similar among treatments, independent of soil conditions and neighboring species. In the control soil, the roots Al concentration of *G. avellana* plants was significantly lower when this species grows together with a conspecific species than when it grows together with *V. corymbosum.* The Al supplemented soil increased significantly S levels in leaves and roots of both species (**Tables 3**, **4**).

## Discussion

Contrary to initial expectations, there was no evidence of the facilitating effect of *G. avellana* on the *V. corymbosum* seedlings growth (**Figure 1**). Rather surprisingly, *G. avellana* exhibited an enhancement in its own growth when grown alongside with *V. corymbosum* (**Figure 1**; **Supplementary Table S1**). These findings align with the observations made by Fajardo and Piper (2019), who reported that *Nothofagus* species did not experience an improvement in their survival and growth when they were planted next to *G. avellana*. Notably, however, *G. avellana* did exhibit growth enhancement when co-cultivated with *Nothofagus* or some conspecifics species. These authors concluded that Proteaceae species have a competitive advantage over non-cluster root-bearing species at the seedling stage, especially on nutrient-rich substrates. In our study, it was observed that *V. corymbosum* showed lower roots P concentration when it grows in association with *G. avellana* (**Table 3**). This suggests that the competitive advantage of *G. avellana* may be attributed to the significant exudation of carboxylates such as oxalate and succinate from its large cluster roots (Delgado et al., 2021; Zúñiga-Feest et al., 2021). These exudates likely contributed to the higher leaf P concentration compared with *V. corymbosum* (**Tables 3** and **4**). While there are currently no studies specifically addressing P mobilization around the cluster roots of *G. avellana*, research conducted on other Proteaceae species, such as *Embothrium coccineum* (Delgado et al., 2015; Renderos et al., 2022), have shown similar levels in the labile P fraction in the rhizosphere of mature cluster roots compared to non-cluster roots and bulk soil, hinting at the possibility of localized P mobilization and accelerated P uptake by plants with cluster roots.

Based on the N:P ratio thresholds established by Koerselman and Meuleman (1996), our findings indicate that *G. avellana* consistently experiences co-limitation of both N and P across all treatments. In contrast, the growth of *V. corymbosum* appeared to be predominantly constrained by P, when plants were grown under control soil conditions (**Figure 3**). This observation reaffirms the high P demands of the *V. corymbosum* species, as previously described Pinochet et al. (2014) (≥ 16 mg P kg^-1^ soil). Interestingly, in the Al-supplemented soil, *V. corymbosum* plants were co-limited by N and P. This was attributed to a decrease in foliar N concentration, particularly evident in conspecific plant pairs. The decrease in foliar N concentration due to Al toxicity has been previously reported in *V. corymbosum* (cv. Brigitta) (Qilong et al., 2020). This phenomenon is primarily associated with reduced root growth, resulting in a diminished nutrient uptake ability. In our study, a reduction in roots growth of *V. corymbosum* (**Supplementary Figure S3**) was not observed. It can be speculated that a nutrient imbalance, rather than direct Al toxicity, was most likely the underlying reason for the negative effects of soil Al_2_(SO_4_) supplementation on the foliar N concentration.

Although it was expected to observe a facilitating effect on P acquisition, as in the case of other Proteaceae species inhabiting in extremely P-poor environments (Teste et al., 2020; Shen et al., 2023), a facilitating effect was found for *G. avellana* on *V. corymbosum* in foliar chlorophyll concentration and photosynthesis rate (**Figure 2**, **Table 2**), when both species growth together in Al supplemented soil. Since N is a fundamental element for photosynthetic pigments and consequently for the photosynthesis process (Lambers and Oliveira, 2019), the facilitating effect of *G. avellana* can be attributed to the fact that this species induced a higher N uptake rate in *V. corymbosum* when it grew in Al supplemented soil, presenting values similar to those when it grew in the control soil (**Table 4**). In this context, it is relevant to mention that there is some evidence showing that, unlike other species of Proteaceae species inhabiting in extremely P-impoverished soil (e.g. South Africa and Australia), the southern South American Proteaceae *Embothrium coccineum* induced cluster roots formation mainly under poor N soils, suggesting that this roots structures could promote N–acquisition (Piper et al., 2013). In *Hakea actites* (Proteaceae), the cluster roots formation and expression of peptide transporter were up-regulated in response to N starvation, probably to increase N uptake under conditions of low N availability (Paungfoo-Lonhienne et al., 2009). From these insights, it is proposed that cluster roots of *G. avellana* could indeed possess mechanisms which can facilitate N availability to neighboring plant species. In this regard, one plausible mechanism could involve organic acid release, which stimulate microbial activity in the rhizosphere. Microbial degradation of organic matter can release N, which may subsequently contribute to higher levels of available N for plants. While this hypothesis provides a compelling direction, additional studies are required to reveal the actual role of cluster roots of *G. avellana* in N acquisition and its possible mechanisms underlying this process.

A significantly higher photosynthetic rate was observed in *G. avellana* plants when they were grown in Al supplemented soil, regardless of the plant combinations involved. This intriguing finding is congruent with the growing body of evidence which underscores the benefits associated with the presence of Al in the soil. This is especially pertinent for plant species that are naturally adapted to acidic environments and particularly pronounced in Al hyperaccumulator species (Watanabe and Osaki, 2002; Bojórquez-Quintal et al., 2017; Muhammad et al., 2018; Sun et al., 2020). Similarly, studies have reported higher photosynthetic rates in Al hyperaccumulator plants, such as *Camelia sinensis* and *C. japonica* when were supplied with Al compared to control conditions (Mukhopadyay et al., 2012; Hajiboland et al., 2013; Liu et al., 2020). In *Qualea grandiflora*, another Al hyperaccumulator plant species, a significant increase in photosynthetic pigment concentrations, encompassing chlorophyll a, b, and carotenes, was observed in plants supplied with Al (Cury et al., 2019). Our findings support the contention that Al may exert a positive impact on the photosynthetic rate in the Al-hyperaccumulator plant. In our study, the increase in the photosynthetic rate in *G. avellana* is probably due to the increased stomatal conductance (**Table 2**) and increased proportion of chlorophyll a (**Figure 2C**), the primary chlorophyll pigment for photochemisty (Björn et al., 2009). Furthermore, the increase in leaf Mg concentration (**Table 3**), a central element within the chlorophyll molecule, may also contribute to this effect (Farhat et al., 2016).

In addition to Mg, the concentration of other essential mineral nutrients such as K and Mn significantly increased in both leaves and roots of both species when grown in the Al supplemented soil. This effect held true regardless of whether the neighboring species is interspecific or conspecific. Furthermore, in the Al hyperaccumulator species, *G. avellana*, there was a significant rise in Na levels in leaves; and a concurrent increase in Fe, Cu and Zn in the roots was observed. The beneficial effects of Al on plant nutrient uptake have been observed in other species. For instance, Osaki et al. (1997) reported increased content of N, P, Mg and K in different organs of several plant species upon Al treatment. Similarly, the stimulation of growth by Al in the Al hyperaccumulator *C. sinensis* was attributed to the increased absorption of vital plant nutrients, such as Ca, Mg, K and Mn (Fung et al., 2008), as well as N and P (Konishi et al., 1985). Bojórquez-Quintal et al. (2017) proposed that Al could induce the expression or activity of transport proteins (channels and transporters) and an alteration in the membrane potential and proton (H+) flux. This, in turn, could facilitate nutrients uptake by plant roots.

Carotenoids are known to play important roles in plants as antioxidants, and during environmental stress, plants usually respond by increasing their carotenoid concentrations (Havaux, 2014). Intriguingly, our observations revealed significantly lower concentrations in leaf carotenoids of *G. avellana* plants cultivated in Al-supplemented soil compared to control (**Figure 2E**), suggesting that this species may not have been experiencing stress during Al exposure. Indeed, the lipid peroxidation of *G. avellana* leaves was lower in plants that were cultivated in the Al supplemented soil. Similar results were found in the roots of *C. sinensis*, where it was observed that Al reduced the lipid peroxidation in root tips, which was related to the fact that Al-induced an increase in the activities of antioxidant enzymes (e.g. superoxide dismutase, catalase, and ascorbate peroxidase (Ghanati et al., 2005). Thus, the results suggest a positive effects of Al on the membrane integrity of *G. avellana* plants. On the contrary, higher lipid peroxidation was observed in the leaves of *V. corymbosum* plants that were cultivated in the Al supplemented soil, supporting previous reports indicating membrane damage induced by Al in this species (Ulloa-Inostroza et al., 2016; González-Villagra et al., 2021). Interestingly, *V. corymbosum* accompanied by *G. avellana* reduced significantly the lipid peroxidation in leaves when grew in the Al supplemented soil, clearly showing a facilitating effect of *G. avellana* towards *V. corymbosum*. It is proposed that, by increasing the N concentration of *V. corymbosum* when it grew with *G. avellana* (**Table 4**), *V. corymbosum* could have increased the activities of antioxidant enzymes, thus decreasing lipid oxidation.

In both species, leaf Al concentration was similar among treatments, suggesting a high self-regulation in Al uptake. This adaptive mechanism is highlighted by Delgado et al. (2019), who found that *G. avellana* growing in natural conditions showed similar leaf Al concentrations, independent of Al saturation percentage and soil pH. These authors reported ranges from 3,959 to 6,256 mg Al kg^-1^ for mature leaves and 7,150 to 11,040 mg Al kg^-1^ for senescent leaves of adult plants. In contrast, the current study used of 2-year-old plants, with an average of 1,505 mg Al kg^-1^. It is most probable that the capacity for Al hyperaccumulation in this species, increases with the age of the plant. With respect to roots Al concentration, *G. avellana* plants exhibit significantly higher values when grown alongside *V. corymbosum*, compared to cultivation alongside conspecific species in soil without Al supplementation (**Table 3**). It was found that in this soil condition, leaves of *V. corymbosum* were limited by P (**Figure 3**) and therefore, it is likely that *V. corymbosum* activated mechanisms such as roots carboxylate exudation to mobilize P from the soil. Such responses are common among plants facing P deficiency (Chen and Liao, 2016). Beyond liberating P from the soil, it also serves as a formidable defense against Al toxicity, particularly in plants that possess the inherent ability to exclude the entry of Al into their tissues. This is achieved through the formation of harmless Al-organic complexes within the rhizosphere, reducing the potential harm posed by Al (Kochian et al., 2004; Chen and Liao, 2016). Thus, it is proposed that the greater Al uptake of *G. avellana* was due to the fact that it took up the Al excluded by *V. corymbosum*. Interestingly, *V. corymbosum* presented higher Al concentrations in the roots than in leaves (36 to 69 times more), a pattern observed in Al-sensitive plants (Kochian et al., 2004; Alarcón-Poblete et al., 2020; Cárcamo-Fincheira et al., 2023). This trend underscores a limited translocation of Al, thus, confirming the low Al tolerance in the aerial part of the *V. corymbosum* (Star cultivar) plant.

When plants were grown in Al supplemented soil, both species increased their Mn uptake. This could be due to Al supplementation decreasing the soil pH from 6.1 to 4.7 (**Table 1**) thereby increasing the solubility of Mn with decreasing soil pH (Lambers and Oliveira, 2019). On the other hand, several authors have reported that leaf Mn concentration is a good proxy for rhizosphere carboxylate concentrations (Shane and Lambers, 2005; Lambers et al., 2015b; Pang et al., 2018; Lambers et al., 2021). This is because carboxylates exuded by roots simultaneously mobilize P and other nutrients from the rhizosphere, especially Mn, which enters roots via transporters with limited substrate specificity (Lambers et al., 2021). Indeed, the leaf Mn concentration in 100 chickpea genotypes has been positively correlated with carboxylate amount in rhizosheath (Pang et al., 2018). In the current study, there was a 2-fold increase in foliar Mn concentration in *G. avellana* when grown in Al supplemented soil, while in *V. corymbosum* this increase was 7-fold, which was independent of the neighboring species. These results suggest that *V. corymbosum* presented high stress in the Al-supplemented soil and it is likely that this species may have exuded large amounts of carboxylates under these conditions.

The S values were exceptionally high in the soil supplemented with Al_2_(SO_4_)_3_ compared with those values found in the soil of control treatment (**Table 1**). Consequently, leaf S concentrations of *G. avellana* and *V. corymbosum* plants supplemented with Al_2_(SO_4_)_3_ were up to 12 and 15 times more than control plants (**Tables 3**, **4**), with these values being considered in the high ranges for plants (~ 10 mg kg^-1^ DW) (Lambers and Oliveira, 2019). Sulfur is integral to numerous physiological processes in plants, including protein synthesis, enzyme activation, chlorophyll formation, defense mechanisms, and secondary metabolite production (Kopriva et al., 2019). It has been reported that S can alleviate the toxicity of metals such as cadmium (Ferri et al., 2017; Lyčka et al., 2023) and Al (Guo et al., 2017), because some S-containing compounds, such as glutathione and phytochelatins, play vital roles in the complexation of metals and their subsequent sequestration into vacuoles. Additionally, these S-containing compounds contribute to the alleviation of oxidative stress by functioning as antioxidant molecules. Our results showed that the addition of Al_2_(SO_4_)_3_ seems to benefit *G. avellana*, due to increased proportion of chlorophyll a (**Figure 2C**), photosynthetic rate (**Table 2**), leaf Mg concentration (**Table 3**) and decreased lipid peroxidation (**Figure 4**). On the contrary, *V. corymbosum* plants worsens their condition under the soil supplemented with Al_2_(SO_4_)_3_, raising the possibility that S supplementation may not have been sufficient to alleviate Al toxicity or that the S could have generated a toxic effect on the plant. Most studies have focused on the interactions occurring between S and other macro and micro-nutrients when plants are subjected to S deficiency (Courbet et al., 2019), however, the excess of S and its effect on the plant has been scarcely explored. We recognize that future studies are necessary to elucidate the effect of Al and S separately on these plants. In any case, it is important to highlight and report that *G. avellana* facilitate to *V. corymbosum* when both species grown in the Al_2_(SO_4_)_3_ supplemented soil. Additionally, it is interesting to mention that unlike *G. avellana*, another Proteaceae species, *Hakea prostrata*, tightly controls its S acquisition (Prodhan et al., 2017). The ability to modulate S and N uptake in some species, has been linked to match its low protein concentration and low demand for rRNA, and its consequently low P requirements (Prodhan et al., 2017), as an adaptation of plants that have evolved in extremely P-impoverished habitats [values of total soil P ranging from 6.6 to 20.3 mg P kg^−1^ (Hayes et al., 2014)]. Thus, it is probable that *G. avellana*, which has evolved on relatively nutrient-rich soils (values of total soil P ranging from 63.1 to 951.6 mg P kg^−1^ (Delgado et al., 2018)), has not evolved the ability to regulate S uptake.

## Conclusions

Contrary to expectations, it was rather surprisingly found that *G. avellana* did not contribute to higher growth rates either P acquisition in *V. corymbosum*. On the contrary, *G. avellana* shows better growth rates when grown associated with *V. corymbosum*. In addition, *V. corymbosum* had a decrease in root P concentrations, when grown in association with *G. avellana*, indicating a highly competitive capacity of this latter species for P acquisition. On the other hand, *G. avellana* did not assist in the decrease of Al uptake of *V. corymbosum*, although it contributed to an increase in N acquisition, and consequently higher chlorophyll concentrations and photosynthesis rates. Besides, *V. corymbosum* decreased the lipid peroxidation in leaves when grown in soil with Al supplementation and accompanied by *G. avellana*. Overall, our results suggest a facilitating effect of *G. avellana* to *V. corymbosum* and *vice versa*. The benefit of facilitation of *G. avellana* to *V. corymbosum* is only observed when both species grown in the Al supplemented soil. The information provided in this manuscript is relevant to know some nutritional and physiological aspects of these species. Future research is required to reveal the possible role of cluster roots of *G. avellana* in the N acquisition. Finally, this study has opened avenues for future research, suggesting the need for additional facilitation tests employing soil with more pronounced contrasts of the studied element (e.g. N, P or Al, and maintaining other soil properties constant) in order to find a greater impact of the nutrient under study on growth of the plant.

## Data availability statement

The raw data supporting the conclusions of this article will be made available by the authors, without undue reservation.

## Author contributions

MD: Conceptualization, Data curation, Formal analysis, Funding acquisition, Investigation, Methodology, Project administration, Resources, Software, Supervision, Validation, Visualization, Writing – original draft, Writing – review & editing. PB: Conceptualization, Funding acquisition, Investigation, Methodology, Supervision, Writing – review & editing. GB: Funding acquisition, Methodology, Writing – review & editing, Formal analysis. ML: Investigation, Methodology, Resources, Writing – review & editing. PD: Investigation, Methodology, Resources, Writing – review & editing. AV: Conceptualization, Supervision, Visualization, Writing – review & editing. MR-D: Investigation, Methodology, Resources, Writing – review & editing.
