# Supplementary material for: Do aluminum (Al)-hyperaccumulator and phosphorus (P)-solubilising species assist neighbouring plants sensitive to Al toxicity and P deficiency?
Source: Front Plant Sci. 2024 Apr 24;15:1371123. doi: 10.3389/fpls.2024.1371123 (PMC11076858; doi:10.3389/fpls.2024.1371123)
Supplement: Supplementary file 1 [file DataSheet_1.docx]

Supplementary Material


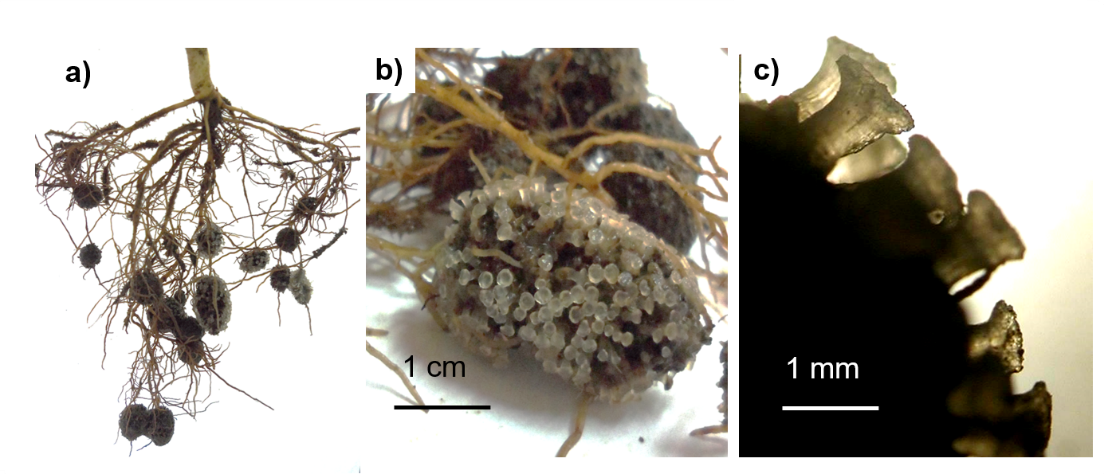


**Supplementary Figure 1** a) Root system of *Gevuina avellana*, b) cluster roots and c) magnified rootlets with “claviform” tips of a mature cluster root.


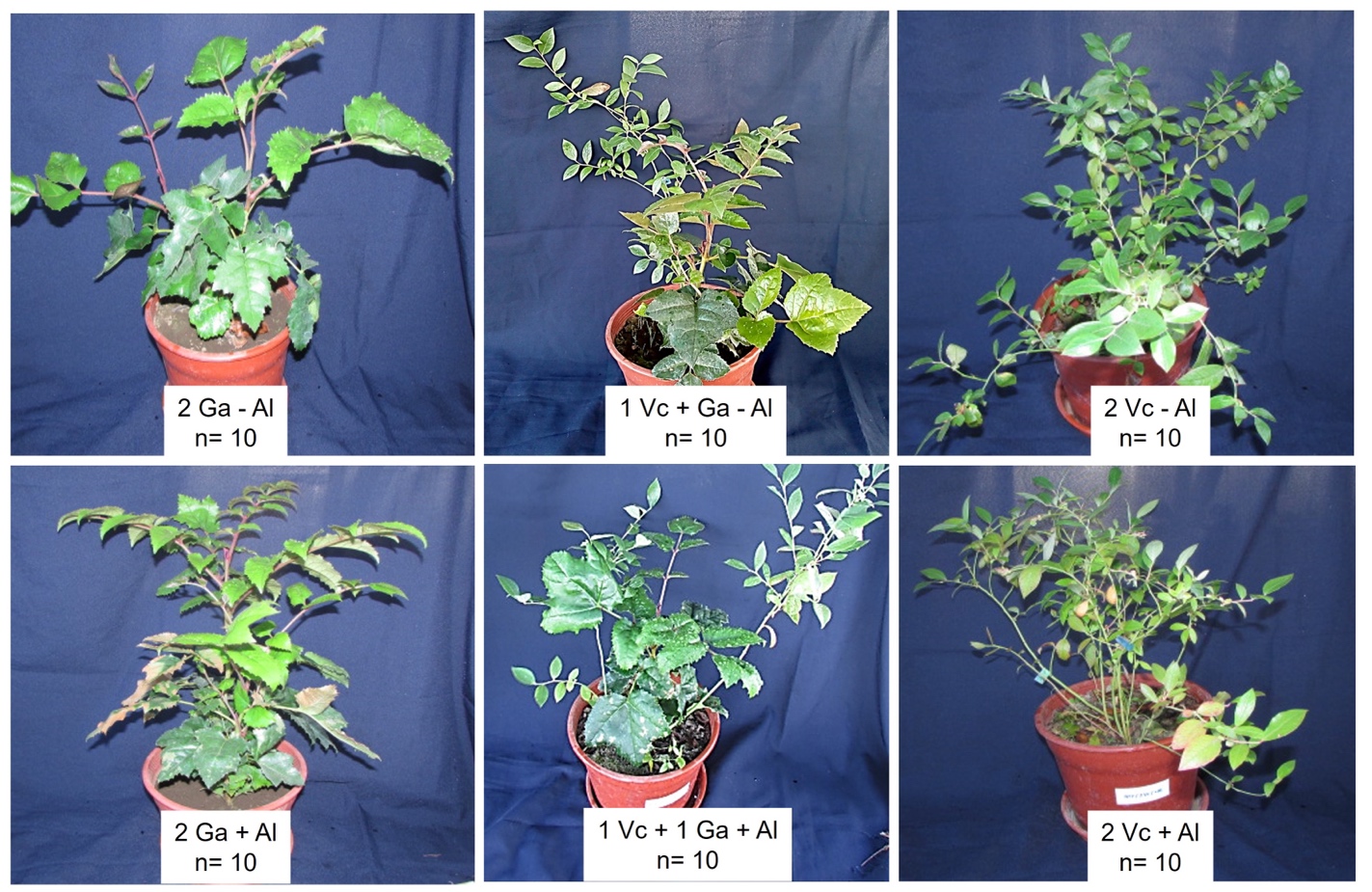
 **Supplementary Figure 2.** Plants of *Gevuina avellana* and *Vaccinium corymbosum* growing alone or in combination with or without aluminum (Al) supplementation (as Al_2_(SO_4_)_3_)) at the end of the experiment.


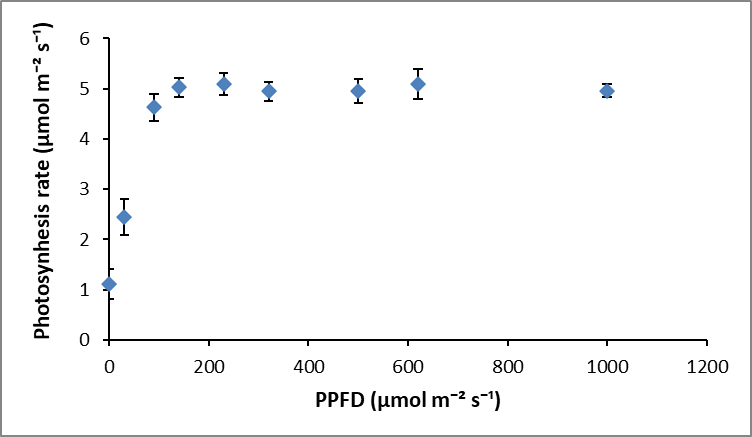


**Supplementary Figure 3.** Light response curves of net photosynthesis rate as a function of photosynthetic photon flux density (PPFD) in mature leaves of *Gevuina avellana growing in greenhouse conditions* (For more details of growth conditions see manuscript). Each value corresponds to a mean of 3 samples ± standard error**.**


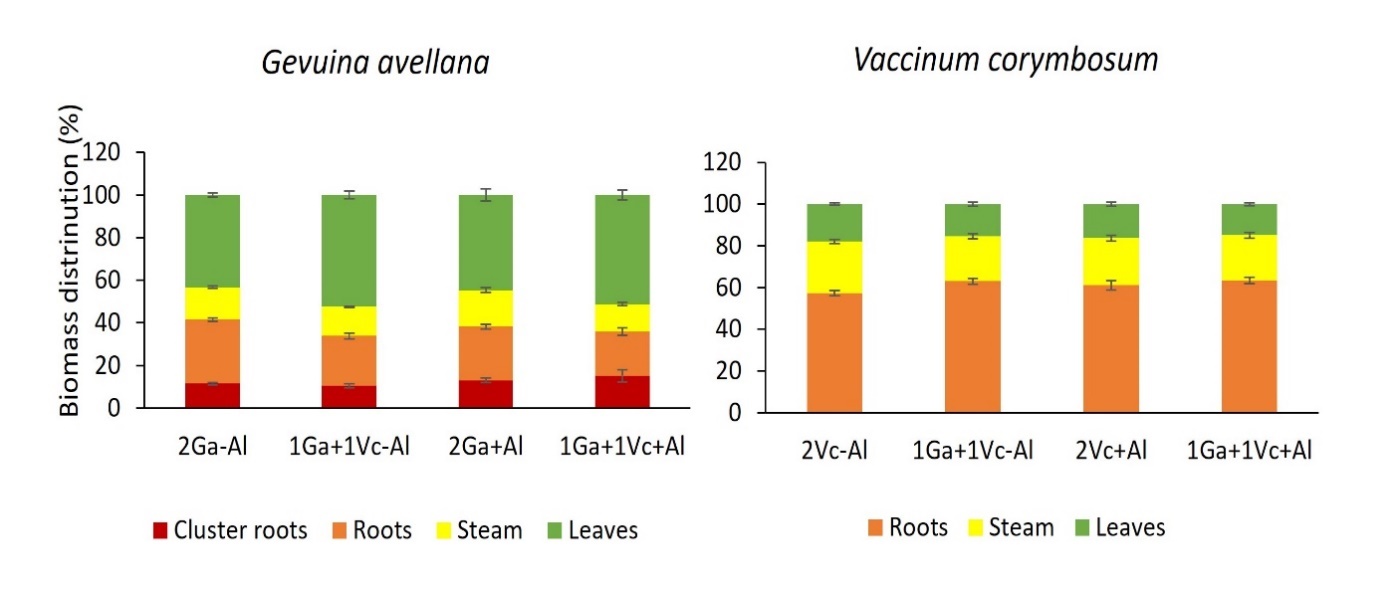
 **Supplementary Figure 4.** Biomass distribution of *Gevuina avellana* and *Vaccinium corymbosum* growing alone or in combination with or without aluminum sulfate (Al_2_(SO_4_)_3_) supplementation.

**Table S1.** Total biomass of *Gevuina avellana* and *Vaccinium corymbosum* growing alone or in combination with or without aluminum sulfate supplementation. Each value corresponds to mean of eight to ten samples ± standard error (SE). Different letters indicate significant differences among treatments for *G. avellana* (*P* ≤ 0.05). n.s.: There are no significant differences among treatments for *V. corymbosum*

| *Gevuina avellana* | |  | *Vaccinium corymbosum* | |
| --- | --- | --- | --- | --- |
| Treatment | Total biomass (g) |  | Treatment | Total biomass (g) |
| 2Ga-Al | 38.9 (2.5) b |  | 2Vc-Al | 32.6 (2.1) n.s. |
| 1Ga+1Vc-Al | 58.2 (3.8) a |  | 1Ga+1Vc-Al | 33.0 (3.9) n.s. |
| 2Ga+Al | 35.0 (2.8) b |  | 2Vc+Al | 31.2 (3.1) n.s. |
| 1Ga+1Vc+Al | 46.0 (4.5) ab |  | 1Ga+1Vc+Al | 31.5 (3.9) n.s. |
